# A comparative analysis of descriptive podcasts vs. interview-based podcasts in enhancing clinical reasoning skills of undergraduate medical students

**DOI:** 10.12669/pjms.42.1.12332

**Published:** 2026-01

**Authors:** Zainab Kamal, Rehan Ahmed Khan, Shabana Ali, Maria Ilyas Khan

**Affiliations:** 1 Zainab Kamal, MBBS, MHPE, Riphah International University, Islamabad, Pakistan; 2 Rehan Ahmed Khan, MBBS, FCPS. MRCS, FRCS, MHPE, PHD-HPE, DEAN RARE Professor of Surgery & Assistant Dean Medical Education, Riphah International University, Islamabad, Pakistan; 3 Shabana Ali, MBBS, MPhil, MHPE, PGD Professor of Anatomy Assistant Dean Medical Education, Riphah International University, Islamabad, Pakistan; 4 Maria Ilyas Khan, MBBS, MHPE, MPhil (Pharma), Riphah International University, Islamabad, Pakistan

**Keywords:** Audio podcasts, Clinical reasoning, Medical education, Technology in learning

## Abstract

**Background & Objective:**

Audio podcasts offer flexibility and align with adult learning principles, yet their role in enhancing clinical reasoning and optimal delivery format remains unclear. This study compared descriptive and interview-based podcasts to evaluate their effectiveness in improving clinical reasoning in undergraduate medical students and determine the superior podcast format.

**Methodology:**

The study conducted from March to May 2024 used a pretest-posttest crossover design to evaluate the effectiveness of two podcast formats (descriptive and interview-based) in enhancing clinical reasoning skills among 93 fourth year MBBS students at Islamic International Medical College during their surgery clerkship. Ethical approval was obtained, and participants were randomly divided into two groups (A and B). Four podcasts, approximately 10 minutes each, were created on acute appendicitis and acute cholecystitis. In Round-1, a pretest on acute appendicitis was followed by Group-A listening to a descriptive podcast and Group-B to an interview-based podcast, with a posttest conducted afterward. In Round-2, the groups crossed over for acute cholecystitis, followed by a posttest. Data were analyzed using SPSS, with paired t-tests comparing pretest and posttest scores within groups and independent t-tests comparing performance between formats.

**Results:**

Both groups demonstrated notable increases in knowledge. In Rounds 1 and 2, Group-A’s mean pretest/posttest score differences were 4.33 and 6.91, respectively, but Group-B were 8.00 and 6.97 (p<0.001). The advantage of the interview-based approach was demonstrated by independent sample t-tests (p<0.001).

**Conclusion:**

Clinical reasoning abilities can be improved with the use of podcasts. Compared to the descriptive style, the interview-based format was more successful, indicating that it may be used as an additional learning aid in medical education.

## INTRODUCTION

By offering cutting-edge resources that improve teaching and learning, the use of technology into medical education has revolutionized conventional learning paradigms.^1^ Because of its adaptability, accessibility, and compatibility with adult learning concepts (andragogy), audio podcasts have become a popular media.^2^ Podcasts assist self-directed learning in medical education by giving students an easy method to interact with difficult subjects outside of traditional classroom settings. Even while podcasts are becoming more and more popular, nothing is known about the best format for fostering critical abilities like clinical reasoning.^3^ As undergraduate medical students enter clinical practice, clinical reasoning which refers to the capacity to evaluate clinical data and reach wise medical conclusions-becomes crucial.^4^ It is essential to improve these abilities using efficient teaching resources, and audio podcasts provide a viable path.^5^

Heutagogy, which emphasizes self-determined and autonomous learning, is particularly relevant in the context of podcasts. It empowers learners to take control of their educational journey, making decisions about what, when, and how they engage with content.^6^ This self-directed approach complements the flexibility offered by podcasts, allowing students to deepen their understanding at their own pace.^7^ Podcasts have the potential to bridge the gap between theory and practice by providing expert insights and examples that foster critical thinking. By incorporating andragogical and heutagogical principles, podcasts offer a valuable tool for enhancing clinical reasoning skills in medical education.^8^

Despite growing evidence supporting the use of auditory podcasts as a learning tool, there is limited research on their effectiveness in teaching complex skills like clinical reasoning, problem-solving, and critical thinking. While podcasts have been shown to enhance basic understanding and knowledge dissemination, the role they play in developing these advanced skills has not been thoroughly explored. Additionally, there is a lack of studies investigating the preferred podcast formats, such as interview-based or descriptive podcasts, and their effectiveness in engaging learners. Addressing these gaps could provide valuable insights into how educators can optimize podcast-based education for better learning outcomes.

The study aimed to evaluate how well descriptive and interview-based podcasts help undergraduate medical students develop their clinical reasoning abilities.

## METHODOLOGY

To evaluate the effects of two podcast formats on clinical reasoning, a quantitative pretest-posttest crossover design was employed. Targeting fourth-year MBBS students enrolled in the surgical clerkship module on acute abdominal crises, the study was carried out at Islamic International Medical College in Rawalpindi, Pakistan. The study was conducted from March 2024 to May 2024.

### Ethical approval:

The Institutional Review Committee of Riphah International University’s Islamic International Medical College granted ethical approval. The head of the institution gave permission to perform the investigation vide (RIPHAH/IRC/24/1038; dated: February 12, 2024).

### Study population and Sample size:

There were 102 fourth-year MBBS students in the target population. A sample size of 87 with a 95% confidence interval and 80% power was determined using OpenEpi; to accommodate for dropouts, the sample size was raised to 100. Ninety-three willing students participated in the final research and were split into two groups (A and B) randomly through lottery method (drawing of lots) to ensure unbiased and equal group allocation.

### Inclusion and Exclusion Criteria:

In order to concentrate on students with advanced clinical reasoning skills, first- to third-year MBBS students were eliminated, whereas fourth-year students were included.

### Data Collection Instrument and Procedure:

In both pre-test and post-test forms, 15-item Extended Matching Questions (EMQs) were used to evaluate clinical reasoning. By simulating authentic clinical situations, EMQs reduced guesswork. Six surgical subject-matter experts validated these inquiries. Before being distributed, two sets of standardized podcasts on cholecystitis and acute appendicitis were created, tested, and verified. These topics were chosen deliberately after consultation with the subject expert as they represent common conditions of comparable complexity and diagnostic reasoning requirements at undergraduate level. The two podcast formats chosen were descriptive and interview based which were played in supervised sessions to ensure uniform exposure and prevent information sharing among groups. Before and after each session, students finished the EMQs and listened to the podcasts.

### Validity and Reliability:

By having EMQs and podcasts validated by experts, the study guaranteed content validity. The alignment of assessment instruments with anticipated clinical competence strengthened construct validity. Bias was reduced by randomly assigning participants and blinding them to the podcast formats. Internal consistency was ensured by allowing each group to sample both podcast kinds through the cross-over concept. Reliability was enhanced by pretesting the intervention materials and data collection instruments. Reliability of the formulated EMQ calculated through Cronbach alpha was found to be 0.7. All treatments were consistent because of standardized recording and delivery procedures.

### Data analysis procedure:

SPSS version 26 was used to analyze the data. Independent t-tests evaluated differences between groups, whereas paired t-tests compared pre- and post-test results within groups. Statistical significance was defined as a p-value of less than 0.05.

## RESULTS

At Islamic International Medical College, 68 (67%) of the 102 fourth-year MBBS students were female, while 34 (33%) were male. This study involved 93 fourth-year MBBS students from Islamic International Medical College, of whom 34 (33%) were male and 68 (67%) were female. Ninety-three students were enrolled during the recruiting period, which ran from April 23 to April 30, 2024. Group-A consisted of 45 students who listened to a descriptive podcast, while Group-B consisted of 48 students who listened to an interview-based podcast. 88 students took part in Round-2, with 45 in Group-B and 43 in Group-A. A pre-test was administered to both groups, followed by an intervention that involved audio podcasts in various formats, and a post-test to assess learning results. Table-I below provides a summary of the pre-test and post-test outcomes for both groups.

**Table-I:** Pre-Test and Post-Test Scores for Group-A and Group-B.

Group	Pre-Test Score (Mean ± SD)	Post-Test Score (Mean ± SD)	Mean Difference (Mean ± SD)	p value
Group-A	8.04 ± 2.80	12.38 ± 2.43	4.33 ± 2.36	< 0.001
Group-B	5.23 ± 2.68	13.23 ± 1.28	8.00 ± 2.76	< 0.001

While Group-B showed a more significant gain of 8.00 points (from 5.23 to 13.23), Group-A’s mean score increased by 4.33 points (from 8.04 to 12.38). Regardless of format, the treatments had a favorable effect on clinical reasoning abilities, as both groups showed statistically significant gains (p < 0.001). The greater improvement seen in Group-B raises the possibility that the podcast format based on interviews was more successful. To evaluate how the treatments affected learning outcomes, paired samples t-tests were used for intragroup comparisons between Group-A and Group-B. Table-II below shows the outcomes for each group.

**Table-II:** Paired Samples t-Test for Group-A and Group-B.

Group	Measure	Mean ± SD	Mean Difference (M ± SD)	t-Value	p Value	n
Group-A	Pre-Test Score	8.04 ± 2.80				45
	Post-Test Score	12.38 ± 2.43	-4.33 ± 2.35	-12.34	< 0.001	
Group-B	Pre-Test Score	5.23 ± 2.68				48
	Post-Test Score	13.23 ± 1.28	-8.00 ± 2.76	-20.08	< 0.001	

**Fig.1:**
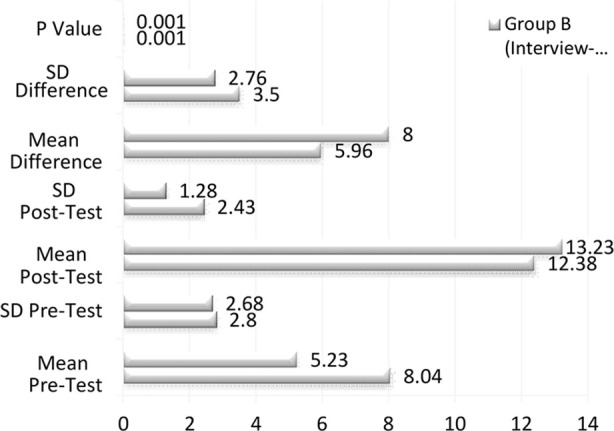
Overall Comparison of Pre-Test and Post-Test Scores.

The pre-test mean score for Group-A was 8.04 (SD = 2.80), and after the intervention, it rose to 12.38 (SD = 2.43), yielding a statistically significant mean difference of -4.33 (SD = 2.35, p < 0.001). With a pre-test mean score of 5.23 (SD = 2.68) and a post-test mean score of 13.23 (SD = 1.28), Group-B also shown a significant improvement, resulting in a mean difference of -8.00 (SD = 2.76, p < 0.001). The usefulness of the audio podcasts in improving clinical reasoning abilities was demonstrated by the considerable improvement in the overall analysis of pre-intervention and post-intervention scores for both groups. The degree of improvement differed across the two podcast formats, though. Fig.1 displays the findings with a statistically significant mean difference of 3.00 points (p < 0.001), comparative analysis showed that Group-B, which employed the interview-based style, improved scores more than Group-A. This implies that the podcast format based on interviews was more successful in improving clinical reasoning abilities. In Round-2, the groups’ intervention formats were varied in order to examine the consistency of the outcomes. Table-III provides a summary of the findings.

**Table-III:** Post-Test Scores After Switching Intervention Formats.

Group	Mean Pre- Test ± SD	Mean Post- Test ± SD	Mean Difference ± SD	p Value
Group-A (Interview-Based Podcast)	7.02 ± 3.10	13.93 ± 1.58	6.91 ± 2.46	< 0.001
Group-B (Descriptive Podcast)	7.84 ± 3.29	13.91 ± 1.84	6.07 ± 3.09	< 0.001

**Fig.2:**
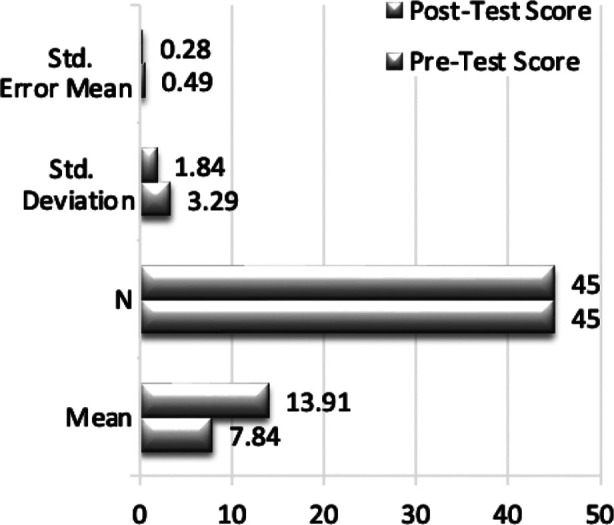
Paired Samples t-Test for Group-B.

The mean pre-test score in Group-A, which was now using the interview-based podcast, was 7.02; after the intervention, it rose to 13.93, resulting in a mean improvement of 6.91 points (p < 0.001). Group-B, which listened to the descriptive podcast, had a mean improvement of 6.07 points (p < 0.001) from their pre-test mean score of 7.84 to their post-intervention mean score of 13.91. As seen in Fig.2 further intragroup-Analysis for Group-B in Round-2 indicated a statistically significant difference between the pre-test and post-test scores. Additional paired samples t-test analysis showed a significantly significant p-value of < 0.001 and a mean difference of -6.07 (SD = 3.09), suggesting that the descriptive podcast intervention improved Group-B’s learning results. Group-A (interview-based podcast) achieved a mean increase of 6.91 points, whereas Group-B (descriptive podcast) improved by 6.07 points, according to a comparison of the pre- and post-test results in Round-2. The mean increases were 0.84 points apart, but this difference was not statistically significant (p = 0.163), indicating that clinical reasoning skills might be improved equally by both podcast formats. Table-IV displays the findings.

**Table-IV:** Overall Comparison of Pre-Test and Post-Test Scores in Round-2.

Group-A	Group-B	Mean Difference	p value
6.91 ± 2.46	6.07 ± 3.09	0.84 ± 0.60	0.163

Finding out whether podcasts would be a better approach to raise clinical thinking abilities was the second aim of the study. The mean score changes between Group-A-interview-based format-and Group-B-descriptive format-were compared using an independent samples t-test. With a mean difference of 0.84 points-not statistically significant (p = 0.163)-there was This implies, as Table-V shows, both forms were equally successful in enhancing learning results.

**Table-V:** Independent Samples t-Test Comparing Podcast Formats.

Test	Mean Difference ± SD	t-Value	p value
Equal variances assumed	0.84 ± 0.60	1.407	0.163
Equal variances not assumed	0.84 ± 0.60	1.414	0.161

## DISCUSSION

With significant increases in both groups, the findings of the study indicate that both descriptive and interview-based podcasts enhanced the clinical reasoning skills of fourth-year medical students. But the interview-based approach produced a more significant increase in knowledge and clinical reasoning ability than the descriptive podcast style. The fact that Group-B had wider pre- and post-test score differences (p<0.001) demonstrated this. These findings reveal that the dynamic and shifting character of podcasts with interviews could appeal to students more and enable deeper learning.

The findings of this study coincide with those of prior studies demonstrating that audio podcasts could assist medical students in applying clinical thought. The results of our study resonate well with the study conducted by Besser et al. who implemented use of podcasts in three different educational context and found podcasts to be a powerful mechanism for teaching in various educational contexts particularly forcing students to think critically, learn on their own and make better judgments.^9^ Both groups in our study considerably raised their clinical reasoning abilities. This implies that regardless of their form, podcast therapies are effective instruments for improving learning conditions. The findings imply that podcasts provide students the option to select how they learn as an additional instrument for education, thereby efficiently bridging the distance between classroom instruction and solitary study.^10^

Learners are more engaged in conversational and engaging podcasts and are increasingly being used by health professionals as demonstrated in a study by Jeffery Ridell et al.^11^, their study focused on the perceptions of health professionals regarding the inclusion of podcasts in broader educational context through a qualitative approach, stressing the importance of usage of podcasts for knowledge translation, Our study positively explored the potential of the audio podcasts for building a core skill - clinical reasoning, the study is one of its kind and would contribute to the continuing landscape of instructional modalities for teaching higher order outcomes.

In this study the gain in knowledge was evident with both formats of podcasts which is in accordance with past research conducted on orthopedics students by Alexander Back et al. conducted on 130 orthopedic residents, comparisons showed that podcast users showed significantly better achievement (p<0.021) as compared to textbook users. They not only demonstrated a superior gain in knowledge but also documented higher satisfaction with the use of audio podcasts as compared to textbook based learning.^12^ With the interview-based podcast style, the learning outcomes were better than with the descriptive podcast format. This back previous studies demonstrating that conversational and dynamic content piques students’ curiosity and drive. Given it sounds like a therapy session, this approach of delivering knowledge might be more practical and understandable. Using these interactive forms-which resemble learning opportunities encountered in clinical settings-past studies have revealed that students are more engaged and better able to recall what they have learnt, Weinstock et al demonstrated the use of audio podcasts on knowledge gain as well as retention in emergency medicine residents.

The study demonstrated the use of audio podcasts by adding interpolated questions noting the gain in knowledge and retention.^13^ The results are similar to our study that produced a notable knowledge gain in undergraduate medical students. Though the interview-based group performed better, it is noteworthy that both strategies resulted in significant learning improvements. This helps to underline how podcasts may be altered to fit various learning environments and yet be a useful teaching tool. Though they might not be as participatory, descriptive podcasts could nevertheless be helpful for students who prefer structured, orderly access to knowledge.^14^ Both groups demonstrated considerable progress, which implies that podcast therapies might be applied to assist medical students who learn differently.

The findings of the study once more highlight the need of giving students’ participation top priority while choosing instructional strategies. Audio podcasts can help in the clinical reasoning skills development through careful integration into the curriculum, as development of this key skill is linked to what, how and when is a subject is taught 15 and audio podcasts can allow strategic yet purposeful designing of curriculum. Incorporating interesting elements into instructional mediums might enable teachers to get their students more interested, a study by Kaplan et al attributed the success of podcasts to its inherent capability to be tailored according to individual need, be it skipping or playing back this asynchronous teaching modality is aligned with the adult learner’s attention span thereby improving their memory and enabling better clinical judgement.^16^

**Chart:**
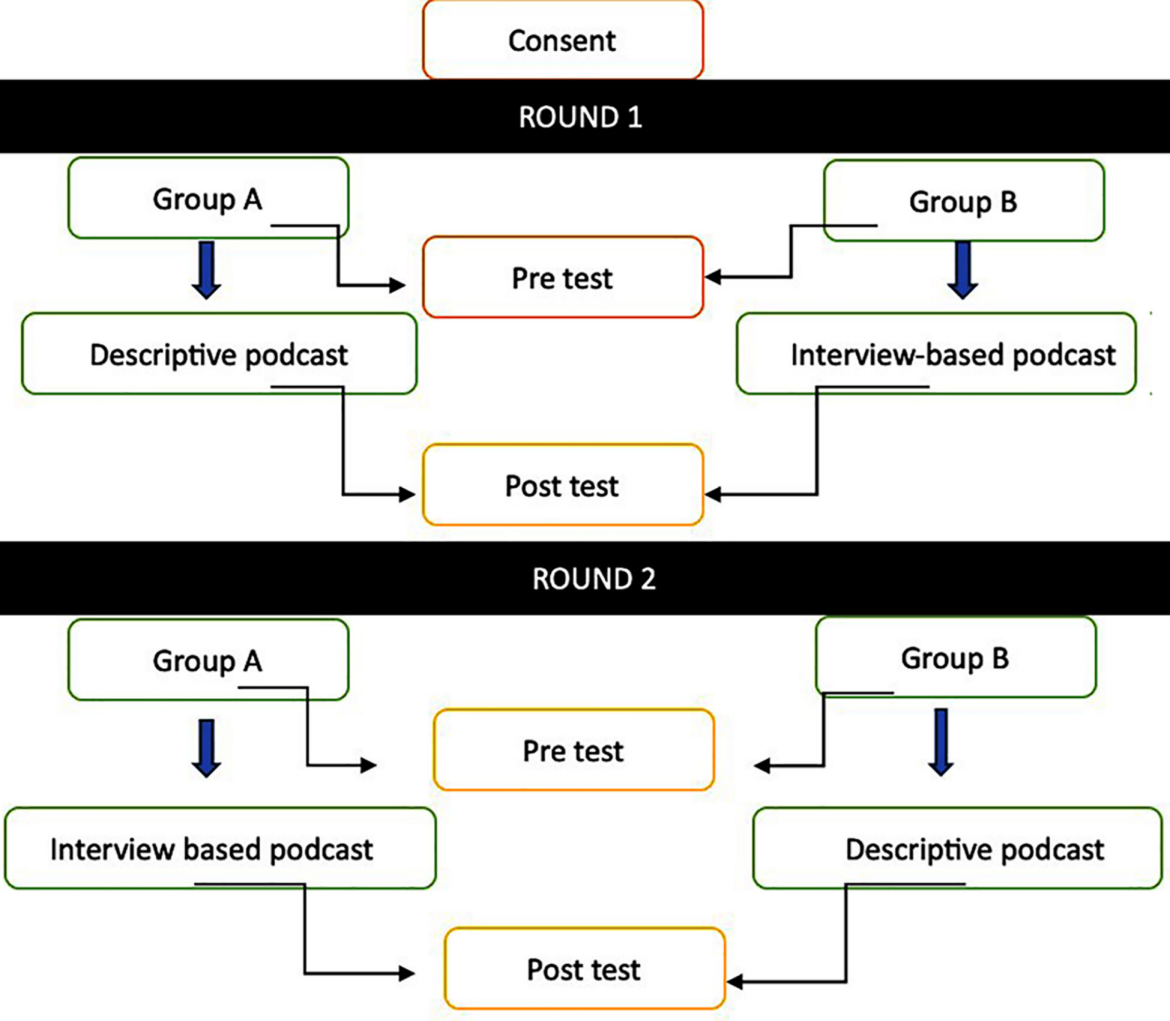
Phases of the study.

At last, the improvement in clinical reasoning abilities points to podcasts as a fantastic tool for teaching students critical medical school skills.^17^ When students are not in a conventional classroom, podcasts provide a simple, flexible, and easily available means for them to interact with challenging content.^18^ This study supports the use of podcast-based therapies in educational environments and ads to the mounting body of data demonstrating their effectiveness. The study design was carefully crafted to explore the use of audio podcasts for achieving higher order outcomes according to Bloom’s taxonomy. In the fast paced, technology integrated world, study material should be made available in all formats to allow learning anytime, anywhere. Our findings offer evidence that audio podcasts can reliably increase clinical reasoning abilities in undergraduate medical students, showcasing the potential for educators to use this modality for instructional purposes.

### Limitations and Future suggestions:

Future research should examine the long-term retention of information following an intervention. Investigating other podcast formats and adding interactive elements might improve this educational tool’s efficacy and level of participation even further.

## CONCLUSION

This study shows that undergraduate medical students’ clinical reasoning abilities are greatly improved by audio podcasts. When comparing the two podcast forms, it was discovered that the interview-based style was more successful in enhancing higher-order outcomes, especially in developing clinical reasoning skills. Audio podcasts support self-directed, lifelong learning and provide students the tools they need to take charge of their education. The interview-based structure is advised for optimizing the educational impact and reaching higher-order outcomes as specified in Bloom’s taxonomy, even if both methods proved advantageous.

### Author`s Contribution:

**ZK and MIK:** Data acquisition, data analysis, drafting the manuscript, critical review.

**RAK and SA:** Study design, data interpretation, critical review.

**ZK and RAK:** Conception, data acquisition, drafting the manuscript,.

All Authors have read and approved the final version and are also accountable for the integrity of the study.
